# Resilience Among Women Living With Infertility: A Cross-Sectional Comparative Study

**DOI:** 10.7759/cureus.109552

**Published:** 2026-05-24

**Authors:** Abdullahi Ibrahim, Anas I Yakubu, Ashiru A Ladan, Abubakar U Mayana, Ibrahim A Umar, Zayyanu Abdullahi, Shamsudeen N Shehu, Amira Bello, Baguda S Abubakar, Ayodeji Bioku, Abbas A Yusuf, Gidado M Ibrahim, Umar F Adamu, Ngozi L Obiejemba, Abdulfattah Isa

**Affiliations:** 1 Psychiatry, Federal Neuropsychiatric Hospital, Kware, NGA; 2 Psychiatry and Behavioral Sciences, Federal Teaching Hospital-Birnin Kebbi, Birnin Kebbi, NGA; 3 Obstetrics and Gynecology, Federal Teaching Hospital-Birnin Kebbi, Birnin Kebbi, NGA; 4 Obstetrics and Gynecology, Usmanu Danfodiyo University Teaching Hospital, Argungu, NGA; 5 Psychiatry, Usmanu Danfodiyo University Teaching Hospital, Argungu, ALB; 6 Psychiatry, Royal Hobart Hospital, Hobart, AUS; 7 Psychiatry, Federal Neuropsychiatric Hospital, Barnawa, NGA; 8 Psychiatry, Nigerian Navy Hospital, Warri, NGA; 9 Psychiatry, Norfolk and Suffolk National Health Service Foundation Trust, Norwich, GBR; 10 Community Health, Federal University-Birnin Kebbi, Birnin Kebbi, NGA

**Keywords:** depression, infertility, infertility-related stress, nigeria, resilience

## Abstract

Background: Resilience constitutes the ability to adapt in the face of adversity, encompassing how women cope with psychological disorders related to infertility. Infertility impacts millions globally, representing a significant reproductive health concern, and has been linked to psychological distress, marital issues, and stigma. However, literature on resilience among women with infertility remains scarce, particularly in Sub-Saharan Africa.

Objective: This study aimed to compare the resilience levels between women with infertility and those who are fertile and to identify the factors associated with low resilience among women experiencing infertility.

Methods: This study was a cross-sectional and comparative investigation conducted at the Usmanu Danfodiyo University Teaching Hospital in Sokoto (UDUTHS). The research included 131 women experiencing infertility and 131 fertile nurses, selected through consecutive sampling over a period of three months. The instruments employed encompassed the 10-item Connor-Davidson Resilience Scale (CD-RISC-10), the Patient Health Questionnaire (PHQ-9), and the Infertility-related Stress Scale (IRSS). Data analysis was performed utilizing IBM SPSS Statistics for Windows, Version 25 (Released 2017; IBM Corp., Armonk, New York, United States). Descriptive statistics were used to summarize the data, and the relationships between variables were examined using the chi-square test.

Results: Infertile women had a higher proportion with low resilience than fertile nurses (76 (58%) vs. 56 (42.7%)). Furthermore, women with infertility had a lower mean resilience score than fertile nurses (26.6 ± 9.6 vs. 29.2 ± 8.1). Lower resilience was significantly associated with tribe (p = 0.018), lower level of education (p = 0.019), shorter duration of marriage (p = 0.021), having no children (p = 0.006), having a nonliving child (p = 0.002), and negative perceptions of adoption. Infertility-related stress (OR = 2.936; 95% CI: 1.088-7.926; p = 0.034) and negative perceptions (OR = 2.812; 95% CI: 1.033-7.659; p = 0.043) remained predictors of low resilience.

Conclusion: Infertility among women is associated with diminished resilience, which is linked to childlessness, depression, and infertility-related stress. It is advisable to integrate routine psychological assessments into fertility clinics to facilitate early identification and evaluation of low resilience. Additionally, provide counseling services to at-risk patients throughout their treatment.

## Introduction

Resilience, seen as a process of ability or as the outcome of successful environmental adaptation, helps people to learn new skills and overcome difficulties while managing the stresses and challenges of everyday life [1]. It is the ongoing process of effectively adapting to challenges and maintaining or restoring psychological well-being. This process serves as a crucial support, helping to buffer the negative impacts of infertility-related stress [2]. Infertile women with good resilience have been reported to have less infertility-related stress, better fertility-related quality of life, and reduced symptoms of depression [3].

Infertility is defined as a condition in which, despite regular unprotected heterosexual intercourse of approximately three to four times per week for a minimum duration of one year, a clinical pregnancy remains unachieved [4-7]. It can be classified as primary if the couple has never conceived; otherwise, it is considered secondary if there was a previous pregnancy [4]. The latter is the most common worldwide among women due to reproductive tract infections [8]. Infertility impacts approximately one in 10 couples worldwide, with a higher prevalence observed among Africans [4,5].

Resilience is neither a fixed personality trait nor an inherent quality but a skill developed over time from various resources, such as strong social networks, adaptive coping strategies, positive religious practices or beliefs, a supportive family, and timely psychosocial interventions when necessary [9]. More resilient people are better equipped to use active and social coping mechanisms, which may serve as a protective factor, lessening the effects of stressors [10]. Resilience seems to reduce anxiety and depression, probably through neurochemical, neuropeptide, hormonal, and genetic means [10].

In vitro fertilization (IVF) is an effective modality of intervention for women with fertility challenges, and among women undergoing IVF for the initial time, higher resilience has been linked to lower stress and depression [11]. However, the protective effect of resilience diminishes or may even be lost completely after one or more unsuccessful IVF outcomes, and the level of stress and depression increases, and resilience no longer appears to buffer them as strongly [11].

Some Iranian scholars compared resilience between fertile and infertile women using a convenience sampling method comprising 80 fertile and 80 infertile women, measured with the Connor-Davidson Resilience Scale (CD-RISC), and found significant differences in resilience, with lower levels in infertile women [1]. Similarly, Sani and colleagues, in their study, found that infertile women demonstrated significantly lower levels of quality of life, self-efficacy, and resilience compared with their fertile counterparts [12]. Furthermore, certain researchers have observed that women experiencing infertility and exhibiting lower resilience levels are subject to increased stress related to infertility. This observation is consistent with prior findings indicating that such stress is associated with a diminished quality of life [13].

Although psychological resilience is regarded as a crucial factor among women in their reproductive years, there is a lack of research examining resilience levels in women with infertility in Nigeria and Sub-Saharan Africa [14,15]. The current study, therefore, sought to determine resilience levels between infertile women and healthy controls and to identify factors associated with resilience among women experiencing infertility.

## Materials and methods

This cross-sectional, comparative study was conducted within the Department of Obstetrics and Gynecology and the Nursing Services at Usmanu Danfodiyo University Teaching Hospital in Sokoto (UDUTHS) over three months, from April to June 2023.

Eligibility criteria

Participants were eligible if they were at least 15 years old (some individuals marry by this age and can provide consent, even though the usual age of consent is 18), fluent in English or Hausa, and willing to participate. Infertile women needed a clinical diagnosis of infertility, confirmed by a gynecologist. Fertile nurses had to be married, have at least one child, and not be receiving fertility treatments. Both groups excluded individuals with severe physical or mental disorders that could impair participation.

Sample size and sampling technique

A study of 156 women with infertility at UDUTH, Sokoto, Nigeria, found a 21.8% depression rate, assessing perception, prevalence, and correlates [5]. A study in Kano, Nigeria, found a 33.7% depression prevalence among female outpatients, examining its prevalence and factors [16], using the following formula:

\[ n = \frac{(Z_{\alpha} + Z_{1-\beta})^2 (p_1q_1 + p_2q_2)}{(p_1 - p_2)^2} \] where: \[ n = \text{minimum sample size} \] \[ Z_{\alpha} = \text{standard normal variate for alpha level of significance at 5\%} = 1.96 \] \[ Z_{1-\beta} = \text{standard normal variate for power of 80\%} = 0.84 \] \[ p_1, p_2 = \text{estimated proportions in the two groups} \] \[ q_1 = 1 - p_1 \] \[ q_2 = 1 - p_2 \]

P1 represents the prevalence of depression among women with infertility from a previous study in Sokoto [5]. P2 represents the prevalence of depression among women at a general outpatient clinic without a history of infertility in Kano from a previous study [16]. Hence, p1 = 21.8% [5] = 0.218; q1 = 1-0.218 = 0.782; p2 = 33.7% [16] = 0.337; and q2 = 1-0.337 = 0.663.

By substitution,

\[ n = \frac{(1.96 + 0.84)^2 \left[(0.218 \times 0.782) + (0.337 \times 0.663)\right]}{(0.218 - 0.337)^2} \]

\[ n = \frac{(2.80)^2 \left[(0.170476) + (0.223431)\right]}{(-0.119)^2} \]

\[ n = \frac{7.84 \times 0.393907}{0.014161} \]

\[ n = \frac{3.08823088}{0.014161} \]

\[ n = 218.08 \]

\[ n \approx 218 \]

The projected number of women experiencing fertility issues who are anticipated to visit the fertility clinic over a six-month period, corresponding to the duration of data collection, is estimated at 260 based on prior records. The population size is less than 10,000. The following formula has been used [17]:

\[ n_f = \frac{n}{1 + \frac{n}{N}} \] where: \[ n_f = \text{estimated sample size} \] \[ n = 218 \] \[ N = 260 \] By substitution: \[ n_f = \frac{218}{1 + \frac{218}{260}} \] \[ n_f = \frac{218}{1 + 0.838} \] \[ n_f = \frac{218}{1.838} \] \[ n_f = 118.61 \] \[ n_f \approx 118 \]

An adjustment was made to increase the sample size to account for the anticipated number of nonresponses or questionnaires that could not be analyzed. The formula for calculating adjusted sample size is as follows [17]:

\[ N = \frac{n}{1-q} \] where: \[ N = \text{adjusted sample size} \]

\[ n = \text{calculated sample size} \]

\[ q = \text{expected nonresponse rate of 10%} \]

By substitution: \[ N = \frac{118}{1-0.1} \] \[ N = \frac{118}{0.9} \] \[ N = 131.1 \approx 131 \] For the two groups: \[ 131 + 131 = 262 \]

Hence, a total of 262 patients participated in the study. Over the three-month study period, participants were consecutively enrolled. A total of 262 eligible participants were recruited, comprising 131 women with infertility and 131 fertile nurses from UDUTHS, ensuring the inclusion of all consenting and accessible patients. 

Data collection procedure and instruments

The study compared mean resilience scores and prevalence rates between the two groups and examined factors associated with resilience among infertile women. The study used several psychometric tools: the Patient Health Questionnaire-9 (PHQ-9) to assess depression, the Infertility-related Stress Scale (IRSS) to assess fertility-related stress, and the CD-RISC to measure resilience, all of which have been used in prior research [18-20]. 

 *Connor-Davidson Resilience Scale (CD-RISC)*

An individual's ability to adapt in the face of adversity is measured using the 10-item CD-RISC scale [21]. This self-administered scale has been employed in Nigeria and is scored on a five-point Likert scale (0 = never to 4 = almost always). It yields a total score ranging from 0 to 40, with 40 indicating the highest level of resilience, calculated by summing responses across components [18]. It possesses favorable psychometric properties, demonstrating very good internal consistency (0.86) and supporting construct validity [21]. As mentioned earlier, the overall score of the CD-RISC scale varies from 0 to 40. Ye et al. suggest that a CD-RISC-10 score below 25.5 indicates a possible concern [22]. Therefore, this research employed a methodology whereby respondents with scores below 25.5 were categorized as having low resilience, whereas those with scores of 25.5 or higher were categorized as possessing high resilience. Additionally, the mean resilience scores and their respective standard deviations were computed for the participants involved in the study

The Infertility-Related Stress Scale (IRSS)

The IRSS is a 12-item self-report tool that measures the impact of infertility on intrapersonal and interpersonal life. It is quick, easy to use, and helps identify whether individuals find interpersonal or intrapersonal functioning more challenging, providing insight into the infertility experience [23,24]. It has two six-item subscales linked to intrapersonal (mental health) and interpersonal (friends) life domains. On a seven-point scale, 1 means "not at all" and 7 means "a great deal"; each item is rated [23].

To determine the intrapersonal domain score, sum items 6, 9, 12, 1, 4, 5, and 6. To calculate the interpersonal domain score, sum items 2, 3, 7, 8, 10, and 11. The overall 12-item score is obtained by aggregating all individual item scores [23]. The total score ranges from 12 to 84, with higher scores indicating greater levels of stress [25]. 

Currently, no validated cutoffs exist for the IRSS. Most studies treat IRSS as continuous, focusing on mean scores, domain differences, and links with anxiety, depression, resilience, and quality of life, emphasizing the mean score [23]. This study used the sample mean as the threshold to classify infertility-related stress. Scores at or above the mean were categorized as high stress, while scores below the mean were considered low stress. This classification served as the basis for calculating the proportion (prevalence) of participants experiencing infertility-related stress.

Patient Health Questionnaire-9 (PHQ-9)

The PHQ-9 is a nine-item self-report questionnaire renowned for its high reliability and robust psychometric properties, and it takes approximately three minutes to complete. It is widely used in clinical, nonclinical, and research contexts [26]. The PHQ-9 accurately captures the reality of depression, with scores ranging from 0 to 27: 5, 10, 15, and 20 denote mild, moderate, moderately severe, and severe depression, respectively. It was used to screen for depression; individuals scoring 5 or above were classified as having depression [26]. It has been used in Nigeria [19].

Data management and statistical analysis

Data were collected, verified, entered, and analyzed with IBM SPSS Statistics for Windows, Version 25 (Released 2017; IBM Corp., Armonk, New York, United States). Descriptive statistics such as percentages, frequencies, and means with standard deviations summarized the data. For bivariate analysis, the chi-square test was used to examine associations between resilience and sociodemographic and clinical variables among women with infertility. A significance level of less than 0.05 was set.

Ethical consideration

The Health Ethics Review Committee at Usman Danfodiyo University Teaching Hospital approved the ethical clearance (UDUH/HREC/2023/1242/V2).

## Results

A total of 262 respondents participated, comprising 131 infertile women attending the fertility clinic at UDUTH and 131 health workers nursing at the same facility.

Level of resilience among the study participants

**Figure 1:**
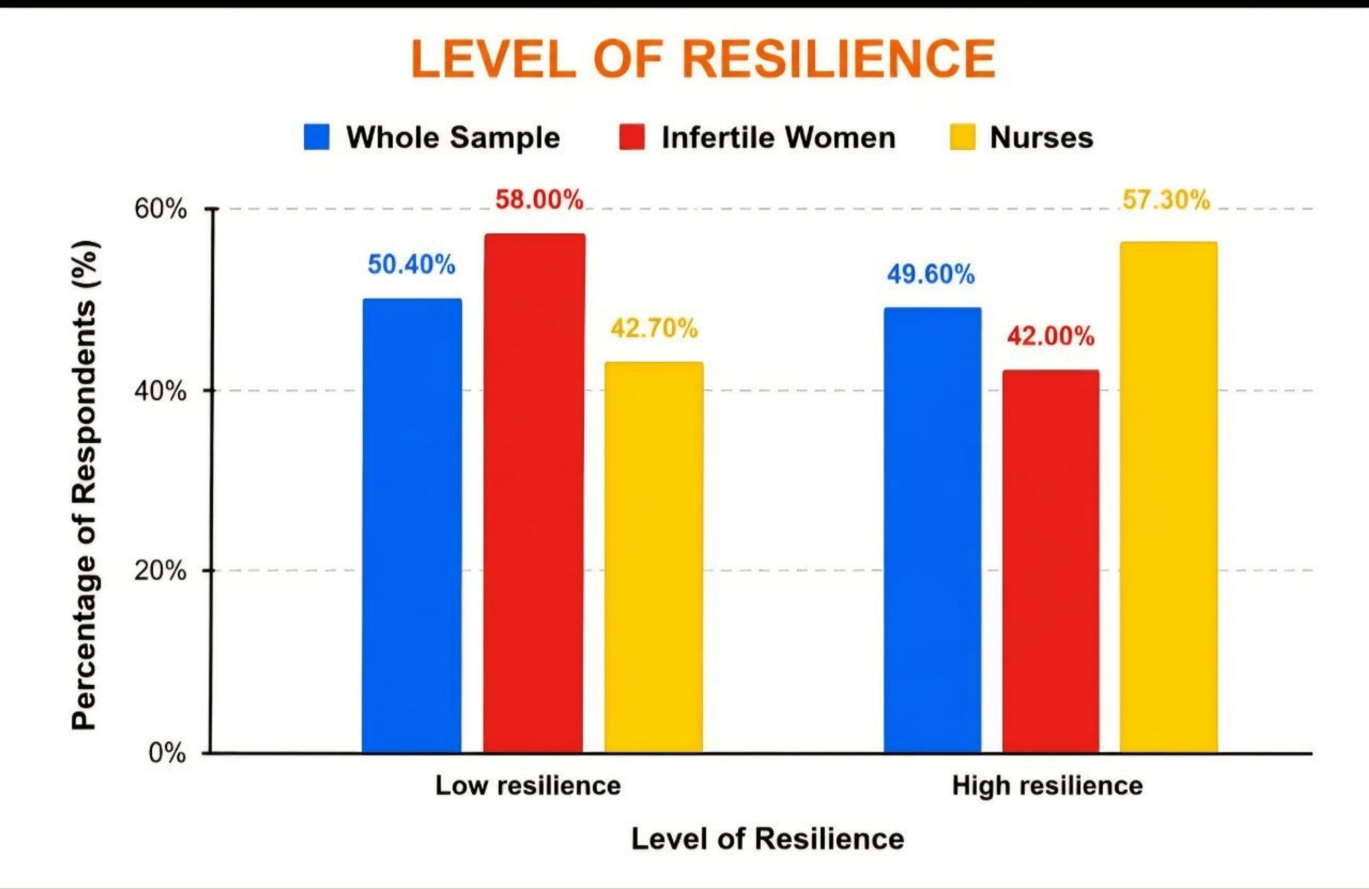
The levels of resilience among the respondents

As shown in Figure 1, a higher proportion of infertile women (58.0%; 95% CI: 49.5%-66.5%) had significantly lower resilience than their fertile counterparts, the nurses (42.7%; 95% CI: 34.2%-51.2%) (χ² = 6.107; p-value = 0.019). Overall, 50.4% (95% CI: 44.3%-56.5%) of respondents demonstrated low resilience. The overall mean resilience score was 17.8 ± 9.0. Infertile women had a lower mean resilience score of 26.6 ± 9.6 than fertile nurses, who had a mean of 29.2 ± 8.1.

Association between sociodemographic factors and resilience among women with infertility

Table 1 below illustrates the association between sociodemographic factors and resilience among women with infertility; infertile women belonging to the other tribe exhibited the lowest levels of resilience, as did those with low levels of education, specifically secondary education and below.

**Table 1:** Association between sociodemographic factors and resilience among women with infertility

Variable	Low resilience n (%)	High resilience n (%)	χ²	df	p-value
Age category (years)			1.356	2	0.508
20-29	42 (59.2%)	29 (40.8%)			
30-39	28 (53.8%)	24 (46.2%)			
40-60	6 (75.0%)	2 (25.0%)			
Tribe			10.012	3	0.018
Hausa	62 (60.8%)	40 (39.2%)			
Yoruba	3 (33.3%)	6 (66.7%)			
Igbo	2 (25.0%)	6 (75.0%)			
Others	10 (83.3%)	2 (16.7%)			
Religion			0.131	1	0.718
Islam	64 (58.7%)	45 (41.3%)			
Christianity	12 (54.5%)	10 (45.5%)			
Level of education			5.530	1	0.019
Below secondary	54 (65.9%)	28 (34.1%)			
Above secondary	22 (44.9%)	27 (55.1%)			
Socioeconomic status			5.919	2	0.052
Low	32 (66.7%)	16 (33.3%)			
Medium	44 (55.0%)	36 (45.0%)			
High	0 (0.0%)	3 (100.0%)			
Family type			2.823	1	0.093
Monogamous	40 (51.9%)	37 (48.1%)			
Polygamous	36 (66.7%)	18 (33.3%)			

Association between marital factors and resilience among women with infertility

Table 2 illustrates the association between marital factors and resilience among women. Those with a shorter duration of marriage were less resilient, as were those who have never had a child, including those with no children alive (even if they have previously delivered) and those who have a negative perception of adoption.

**Table 2:** Association between marital factors and resilience among women with infertility

Variable	Low resilience n (%)	High resilience n (%)	χ²	df	p-value
Marriage duration			5.345	1	0.021
Shorter	62 (63.9%)	35 (36.1%)			
Longer	14 (41.2%)	20 (58.8%)			
Previous marriage			1.510	1	0.219
Yes	30 (65.2%)	16 (34.8%)			
No	46 (54.1%)	39 (45.9%)			
Number of children ever			7.673	1	0.006
None	60 (65.9%)	31 (34.1%)			
At least one	16 (40.0%)	24 (60.0%)			
Alive children			9.852	1	0.002
None	62 (66.7%)	31 (33.3%)			
At least one	14 (36.8%)	24 (63.2%)			
The other wife is having a child (n = 56)			3.733	1	0.053
Yes	36 (66.7%)	18 (33.3%)			
No	0 (0.0%)	2 (100.0%)			
Support from husband			0.406	1	0.524
Yes	68 (57.1%)	51 (42.9%)			
No	8 (66.7%)	4 (33.3%)			
In-law support			1.713	1	0.191
Yes	66 (60.6%)	43 (39.4%)			
No	10 (45.5%)	12 (54.5%)			
Negative perception of adoption			4.616	1	0.032
Yes	22 (45.8%)	26 (54.2%)			
No	54 (65.1%)	29 (34.9%)			

Association between Clinical factors and resilience among women with infertility

Table 3 illustrates the association between clinical factors and resilience among women with infertility. There was a significant association between resilience and the type of infertility, as participants with primary infertility had low resilience, as well as those with high infertility-related stress and those experiencing depression.

**Table 3:** Association between clinical factors and resilience among women with infertility

Variable	Low resilience n (%)	High resilience n (%)	χ²	df	p-value
Duration of infertility			0.731	1	0.393
Shorter duration	4 (44.4%)	5 (55.6%)			
Longer duration	72 (59.0%)	50 (41.0%)			
Type of infertility			14.087	1	<0.001
Primary	54 (72.0%)	21 (28.0%)			
Secondary	22 (39.3%)	34 (60.7%)			
Perceived factor responsible for infertility			1.076	3	0.783
Male	10 (55.6%)	8 (44.4%)			
Female	28 (56.0%)	22 (44.0%)			
Both	6 (75.0%)	2 (25.0%)			
Not sure	32 (58.2%)	23 (41.8%)			
Stress level			15.811	1	<0.001
Low stress	30 (42.3%)	41 (57.7%)			
High stress	46 (76.7%)	14 (23.3%)			
Depression			13.114	1	<0.001
No	50 (49.5%)	51 (50.5%)			
Yes	26 (86.7%)	4 (13.3%)			
Social support			0.135	1	0.713
Poor to moderate	28 (56.0%)	22 (44.0%)			
Good	48 (59.3%)	33 (40.7%)			

Multivariate binary logistic regression

Based on Table 4, variables that were significant in the bivariate analysis were included in a binary logistic regression model. Results showed that infertility-related stress and negative views of adoption were independently associated with low resilience among infertile women, after controlling for other factors. Women with infertility-related stress were about three times more likely to have low resilience (OR = 2.936; 95% CI: 1.088-7.926; p = 0.034). Women with negative perceptions of adoption were about 2.8 times more likely to show low resilience (OR = 2.812; 95% CI: 1.033-7.659; p = 0.043).

## Discussion

The findings of this study indicate that women experiencing infertility exhibit reduced resilience compared to healthy individuals and also present with lower mean resilience scores than the control group. This underscores the understanding that infertility constitutes not merely a reproductive health issue but also encompasses significant psychological dimensions that undermine women's capacity to adapt over time. 

This finding of reduced resilience among women living with infertility was similarly reported by studies conducted internationally, such as in Asia and Europe, which found that infertile women have vulnerabilities, including depression, stress, failed fertility treatments, and impaired emotional adjustment, that may predispose them to low resilience [11,27]. Therefore, the study's findings demonstrated that the pattern observed in Nigeria aligns with those identified in other regions worldwide.

The CD-RISC exists in multiple versions. Comparative analysis of mean scores was challenging; however, we conducted some comparisons across different versions of the scale. The mean resilience score, as measured by the 10-item CD-RISC, in this study was 26.6 ± 9.6. This is lower than the average score reported in the United States (68.1 ± 14.3) [10]. The psychometric tool, i.e., the CD-RISC, exists in three versions: the original 25-item version, the short 10-item version, and the ultra-short two-item form, each with a distinct scoring range. Resilience was assessed in the current study using the 10-item short form, whereas the study conducted in the United States, which reported higher resilience scores, used the original 25-item form [10]. Therefore, the discrepancies in the psychometric instrument may explain the observed differences in mean resilience scores between the two studies. Similarly, another study from Asia that employed the Chinese version of the 25-item scale also reported higher scores than the current study. The authors reported a mean resilience score of 59.53 ± 16.18 [13]. Even though the Chinese version has been validated, differences between versions and languages may explain the variation. An even higher mean resilience score (133.97 ± 26.89) was reported in a study conducted in Pakistan. However, this study employed a different psychometric assessment tool, the Urdu-translated Resilience Scale (RS-1), which has a scoring range of 17 to 75. This methodological difference may account for the discrepancy observed relative to the current study [28].

Various factors have been identified as associated with low resilience among women experiencing infertility, including the absence of children or having no children alive. This may be interpreted within the cultural context where childbirth is highly valued immediately following marriage and childlessness is sometimes regarded as a failure in the matrimonial relationship [13,29]. The study also demonstrates a negative relationship between resilience and depression, aligning with findings observed among Pakistani individuals in the research [28] and similarly supported by Chinese studies [30]. High infertility-related stress was also linked to low resilience. This also aligns with prior studies in Asia [31] and Europe [11]. This is probably because resilience may serve as a moderator to the negative effects of infertility stress, and women with higher resilience tend to have better emotional functioning despite infertility problems [30].

Some participants might have been undergoing medical treatment for fertility issues under the supervision of their gynecologists; therefore, the findings may not be entirely applicable to women who are not seeking such treatment. Furthermore, since the study was conducted within a hospital setting, the results may not be generalizable to the broader population. The psychometric instrument employed to assess reliance, the CD-RISC, was self-administered, which could introduce recall bias and social desirability bias. The study employed a CD-RISC-10 cutoff score of <25.5> to identify low resilience. This threshold was established through validation studies across different cultures and may not be appropriate for Nigerian women experiencing infertility. Cultural differences could influence its applicability. Future research should focus on validating the CD-RISC-10 within the local context and establishing population-specific cutoff points. As the study was cross-sectional, it cannot determine causal relationships and indicates the need for future longitudinal and prospective studies to explore the factors influencing resilience and its long-term effects within this population. It is recommended to routinely incorporate psychological assessments in fertility clinics to evaluate resilience levels and facilitate early identification. Additionally, counseling services should be provided to clients identified as having or at risk of low resilience during infertility treatment. Finally, comparing infertile women with fertile nurses, who differ in education, socioeconomic status, health literacy, healthcare access, coping, and psychological awareness. These differences may cause confounding and independently affect resilience, compromising validity and group comparability. Future studies should use community controls or groups with similar sociodemographics.

## Conclusions

The study found that women living with infertility tend to have lower levels of resilience compared with fertile women. Low levels of resilience were associated with infertility-related stress, depression, not having children, a shorter duration of marriage, and a negative attitude towards adoption. The findings highlight the importance of supporting the psychological well-being of women living with infertility.
